# Effect of calcium dobesilate on macular microvasculature in patients with diabetic retinopathy

**DOI:** 10.1371/journal.pone.0325714

**Published:** 2025-06-10

**Authors:** Jung-Tae Kim, Ka-Hyun Lee, Min-Woo Lee

**Affiliations:** Department of Ophthalmology, Konyang University College of Medicine, Daejeon, Republic of Korea; Dr. Agarwal’s Eye Hospital, INDIA

## Abstract

**Purpose:**

To investigate the impact of calcium dobesilate (CaD) on the macular microvasculature in patients with diabetic retinopathy (DR) using optical coherence tomography angiography.

**Methods:**

In this retrospective study, patients with DR were divided into two groups: those treated with 1 g/day of CaD (Group 1) and those without CaD treatment (Group 2). Following the baseline, patients underwent two additional examinations at 3-month intervals for analysis. The vessel density of the superficial vascular complex (SVD) and deep vascular complex (DVD) were compared against prior assessments. Generalized linear mixed models analyzed factors associated with changes in SVD and DVD over time.

**Results:**

A total of 81 eyes were included: 39 in Group 1 and 42 in Group 2. The mean SVD was 21.8 ± 5.7% at baseline, 23.5 ± 6.5% at 3 months, and 23.3 ± 6.1% at 6 months in Group 1, respectively (P = 0.034), with significant changes observed from baseline to 3 months (P = 0.021), but not from 3 to 6 months (P = 0.745). The mean DVD was 18.2 ± 3.4% at baseline, 20.0 ± 3.9% at 3 months, and 19.9 ± 4.1% at 6 months in Group 1, respectively (P = 0.008), showing a significant increase from baseline to 3 months (P = 0.007), but not from 3 to 6 months (P = 0.825). Group 2 showed no significant changes over time in either SVD (P = 0.175) or DVD (P = 0.156). In Group 1, multivariate analysis identified DR severity as significantly associated with changes in SVD (estimate = 5.07, P = 0.016).

**Conclusions:**

The administration of CaD positively influences macular microcirculation in DR patients, demonstrating its effectiveness even in advanced stages of the disease.

## Introduction

Diabetic retinopathy (DR) is the leading cause of visual impairment among working adults worldwide [1,2]. The pathogenesis of DR involves multiple factors, including the accumulation of sorbitol and advanced glycation end-products, oxidative stress, protein kinase C activation, inflammation, and upregulation of the renin-angiotensin system and vascular endothelial growth factor (VEGF). These factors contribute to vascular endothelial dysfunction, leading to increased vascular permeability, retinal ischemia, and neovascularization [3]. Consequently, retinal vascular changes can lead to complications such as diabetic macular edema (DME), vitreous hemorrhage, and tractional retinal detachment, ultimately resulting in permanent visual impairment. Current therapeutic interventions, such as intravitreal injections of steroids or anti-VEGF agents and pan-retinal photocoagulation (PRP), are invasive and generally reserved for the advanced stages of DR when significant visual impairment has occurred.

Calcium dobesilate (CaD), recognized for its angioprotective properties and beneficial effects on the inner blood-retinal barrier (BRB), is approved for DR treatment in various countries [4]. Clinical trials have shown its efficacy in showing the progression of early DR, attributed to its anti-inflammatory and antioxidant activities, antiangiogenic effects, and enhancement of endothelial-dependent vasodilation [5,6]. Despite these findings, the effectiveness of CaD in DR management remains a subject of debate, as some studies reported no significant difference in the development of DME between patients treated with CaD and those receiving placebo, nor any discernible benefits in retinal imaging outcomes [7,8]. Additionally, no research has assessed the impact of CaD on the macular microvasculature in DR patients as far as we know.

This study aimed to investigate the influence of CaD on the macular microvasculature in DR patients using optical coherence tomography angiography (OCTA).

## Methods

### Patients

This retrospective, observational study adhered to the principles of the Declaration of Helsinki and received approval from the Institutional Review Board/Ethics Committee of Konyang University Hospital, Daejeon, Republic of Korea (No. 2024-03-006). The data for research purposes were assessed on April 12, 2024. Patients who visited our retinal clinic between March 2022 and December 2023 were screened for inclusion. The requirement for informed consent was waived by the Institutional Review Board/Ethics Committee of Konyang University Hospital due to the retrospective nature of the study. Patients with DR were enrolled and divided into two groups: Group 1 consisted of those who had initiated treatment with 1 gram of CaD per day, while Group 2 consisted of patients who had not received a CaD prescription. Following the baseline visit, two additional examinations were conducted at 3-month intervals for analysis. Exclusion criteria included a history of any ocular surgery other than cataract extraction, presence of ocular diseases apart from DR, a history of CaD prescription before the baseline, intraocular pressure (IOP) > 21 mmHg, optic disc pathology, central macular thickness > 350 μm or any cystic changes, and vitreous hemorrhage. Patients requiring PRP or intravitreal injections of anti-VEGF or steroids during the study period were also excluded.

### OCTA analyses

OCTA was performed using a Spectralis OCTA2 device (Heidelberg Engineering, Heidelberg, Germany). The device automatically acquired images of the superficial vascular complex (SVC, from the internal limiting membrane to the inner plexiform layer), the deep vascular complex (DVC, from the inner plexiform layer to the outer border of the outer plexiform layer), and the choriocapillaris (CC, a 20 μm thick layer of the Bruch membrane). The DVC and CC images were analyzed using projection artifact removal mode. OCTA scans measuring 6.0 × 4.5 mm and centered on the fovea were stored. Vessel density (VD) and skeletonized VD were measured with ImageJ software (National Institutes of Health, Bethesda, MD, USA). For analysis, eight-bit images were adjusted using the default threshold settings to differentiate features of interest from the background of segmented grayscale images. Using images thus binarized, the VD and skeletonized VD were quantified by dividing the area occupied by white pixels by the total pixel area. OCTA quality was automatically assessed by the inbuilt software, ranging from 0 (no signal) to 40 dB (excellent). Images compromised by fixation or fovea centration loss, motion artifacts, segmentation errors, or OCTA quality < 30 dB were excluded. If both eyes of a patient met inclusion criteria, the eye with superior image quality was chosen for accurate analyses.

### Statistical analyses

Baseline demographics were compared using an independent t-test. To compare the VD of the SVD (SVD), DVC (DVD), and CC (CVD) with previous measurements, paired t-tests with Bonferroni correction were used. Linear mixed models were used to identify significant VD changes over time, incorporating age, sex, IOP, spherical equivalent, best-corrected visual acuity (BCVA), hypertension, DR severity (nonproliferative or proliferative), DR duration, follow-up length, and OCTA quality as fixed effects. A random intercept was included at the eye level. Generalized linear mixed models were used to determine factors influencing changes in SVD and DVD over time in patients taking CaD. Statistical analyses were performed using SPSS statistics software (version 18.0; IBM Corp., Armonk, NY, USA).

## Results

### Demographics

A total of 81 eyes were included: 39 in Group 1 and 42 in Group 2 (Table 1).

**Table 1:** Baseline demographics.

	Group 1 (n = 39)	Group 2 (n = 42)	P-value
Age (year, mean ± SD)	64.4 ± 9.1	64.12 ± 8.7	0.922
Sex (male, %)	14 (35.9)	22 (52.4)	0.136
Laterality (right, %)	20 (51.3)	21 (50.0)	0.908
HbA1c (%, mean ± SD)	7.3 ± 1.5	7.2 ± 1.6	0.792
Smoking (n, %)	6 (15.4)	5 (11.9)	0.574
Hypertension (n, %)	17 (43.6)	12 (28.6)	0.159
SBP (mmHg, mean ± SD)	114.5 ± 9.2	113.5 ± 8.9	0.703
DBP (mmHg, mean ± SD)	73.2 ± 8.0	71.5 ± 8.7	0.305
Lens status (phakic, %)	19 (48.7)	18 (42.9)	0.951
DR duration (year, mean ± SD)	14.1 ± 8.7	13.1 ± 7.8	0.586
DR stage (PDR, %)	20 (51.3)	14 (33.3)	0.102
SE (diopter, mean ± SD)	−0.27 ± 1.28	−0.53 ± 0.90	0.275
IOP (mmHg, mean ± SD)	16.5 ± 3.2	15.4 ± 3.1	0.131
Axial length (mm, mean ± SD)	23.4 ± 1.2	23.6 ± 0.6	0.604
BCVA (logMAR, mean ± SD)	0.09 ± 0.10	0.06 ± 0.13	0.340
CMT (μm, mean ± SD)	287.6 ± 33.7	278.7 ± 28.4	0.180

SBP, systolic blood pressure; DBP, diastolic blood pressure; DR, diabetic retinopathy; PDR, proliferative diabetic retinopathy; SE, spherical equivalent; IOP, intraocular pressure; BCVA, best-corrected visual acuity; CTM, central macular thickness.

At baseline, there were no statistically significant differences between the two groups in terms of age, sex, or DR stage. Blood pressure measured at each visit remained stable in both groups. The mean BCVA was 0.09 ± 0.10, 0.10 ± 0.10, and 0.07 ± 0.10 in Group 1 (P = 0.280), and 0.06 ± 0.13, 0.05 ± 0.10, and 0.05 ± 0.09 in Group 2 (P = 0.247) at baseline, 3 months, and 6 months, respectively. The mean OCTA quality was 32.5 ± 2.5, 32.9 ± 2.2, and 32.9 ± 2.2 dB in Group 1 (P = 0.473), and 33.0 ± 2.3, 32.8 ± 2.2, and 32.8 ± 2.3 dB in Group 2 (P = 0.690) at baseline, 3 months, and 6 months, respectively.

### SVD, DVD, and their skeletonized forms in each visit

The mean SVD was 21.8 ± 5.7, 23.5 ± 6.5, and 23.3 ± 6.1% in Group 1 (P = 0.034), and 22.3 ± 6.6, 22.6 ± 7.3, and 21.4 ± 6.7% in Group 2 (P = 0.175) at baseline, 3 months, and 6 months, respectively (Table 2).

**Table 2:** Optical coherence tomography angiography parameters at each visit.

	Group 1	Group 2	^*^P-value
SVD			
Baseline	21.8 ± 5.7	22.3 ± 6.6	0.751
3 months	23.5 ± 6.5	22.6 ± 7.3	
6 months	23.3 ± 6.1	21.4 ± 6.7	
^†^P-value	**0.034**	0.175	
SKSVD			
Baseline	7.7 ± 2.1	7.5 ± 2.4	0.768
3 months	8.3 ± 2.4	7.5 ± 2.5	
6 months	8.3 ± 2.4	7.0 ± 2.3	
^†^P-value	**0.021**	**0.028**	
DVD			
Baseline	18.2 ± 3.4	18.7 ± 5.8	0.633
3 months	20.0 ± 3.9	18.4 ± 6.1	
6 months	19.9 ± 4.1	17.8 ± 6.4	
^†^P-value	**0.008**	0.156	
SKDVD			
Baseline	7.7 ± 1.3	7.9 ± 2.2	0.548
3 months	8.4 ± 1.6	7.8 ± 2.4	
6 months	8.3 ± 1.6	7.5 ± 2.4	
^†^P-value	**0.020**	0.075	
CVD			
Baseline	26.1 ± 4.3	26.0 ± 3.5	0.924
3 months	26.7 ± 4.6	26.0 ± 4.3	
6 months	27.3 ± 4.1	26.6 ± 4.3	
^†^P-value	**0.033**	0.331	

SVD, vessel density of superficial vascular complex; SKSVD, skeletonized SVD; DVD, vessel density of deep vascular complex; SKDVD, skeletonized DVD; CVD, vessel density of choriocapillaris.

Values in boldface (P < 0.050) are statistically significant.

* Independent t-test for baseline values.

† Linear mixed model.

**Fig 1:**
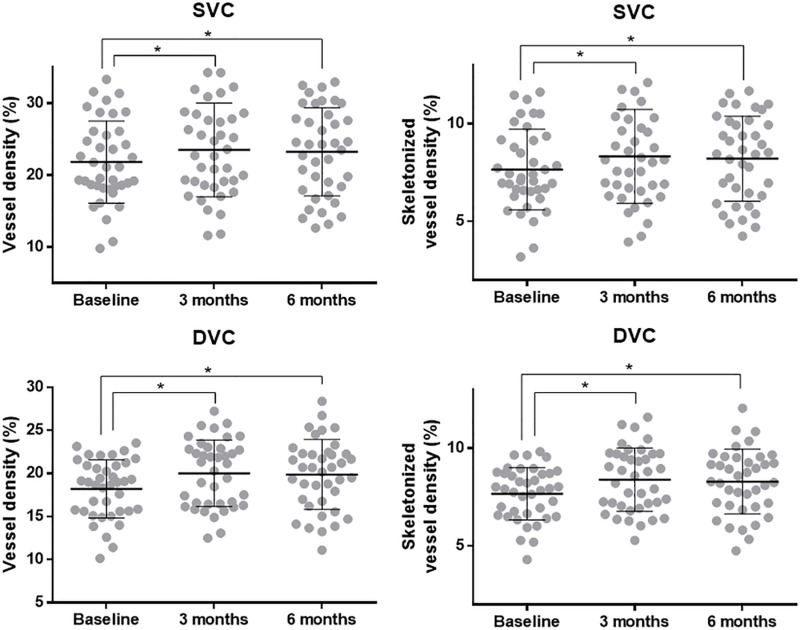
Scatter plots showing the means and standard deviations of vessel densities and skeletonized vessel densities in superficial vascular complex (SVC) and deep vascular complex (DVC) at each visit in patients taking calcium dobesilate. ^*^Statistically significant differences.

In Group 1, there was a significant difference in SVD from baseline to 3 months (P = 0.021), but not from 3 to 6 months (P = 0.745). The skeletonized SVD (SKSVD) also showed significant changes over time (P = 0.021), with pairwise comparison results similar to those of SVD (baseline vs. 3 months, P = 0.018; 3 months vs. 6 months, P = 0.720) (Fig 1). In Group 2, there was no statistically significant change in SVD over time (P = 0.175), while SKSVD demonstrated a significant reduction (P = 0.028).

In Group 1, the mean DVD was 18.2 ± 3.4, 20.0 ± 3.9, and 19.9 ± 4.1% at baseline, 3 months, and 6 months, respectively, indicating a significant change over time (P = 0.008). There was a significant difference in DVD from baseline to 3 months (P = 0.007), but not from 3 to 6 months (P = 0.825). The skeletonized DVD (SKDVD) revealed significant changes over time (P = 0.020), with pairwise comparison results similar to those of DVD (baseline vs. 3 months, P = 0.008; 3 months vs. 6 months, P = 0.723). In Group 2, there was no statistically significant change in DVD (P = 0.156) or SKDVD (P = 0.075) over time.

In Group 1, the mean CC was 26.1 ± 4.3, 26.7 ± 4.6, and 27.3 ± 4.1% at baseline, 3 months, and 6 months, respectively, demonstrating a significant change over time (P = 0.033). However, the pairwise comparisons were not statistically significant (baseline vs. 3 months, P = 0.326; 3 months vs. 6 months, P = 0.318). In Group 2, there was no statistically significant change in CVD (P = 0.331) over time.

### Associated factors for SVD and DVD changes in patients taking CaD

In univariate analyses, age (estimate = −0.40, P < 0.001), DR severity (estimate = 7.14, P < 0.001), and time (estimate = 0.76, P = 0.034) were significant factors associated with changes in SVD (Table 3). Multivariate analyses showed that DR severity (estimate = 5.07, P = 0.016) and time (estimate = 0.73, P = 0.009) remained significant.

**Table 3:** Linear mixed-effect model determination of factors associated with vessel density changes in patients taking calcium dobesilate.

	SVD				DVD			
	Univariate		Multivariate		Univariate		Multivariate	
	Estimate (95% CI)	P value	Estimate (95% CI)	P value	Estimate (95% CI)	P value	Estimate (95% CI)	P value
Age	−0.40 (−0.57, −0.23)	**< 0.001**	−0.14 (−0.38, 0.09)	0.221	−0.14 (−0.24, −0.04)	**0.008**	−0.07 (−0.20, 0.07)	0.322
Sex	−0.89 (−4.73, 2.94)	0.639			0.26 (−1.73, 2.24)	0.795		
Hypertension	2.84 (−0.77, 6.46)	0.120			0.86 (−1.05, 2.77)	0.366		
Smoking	−1.13 (−3.27, 1.01)	0.301			−2.01 (−4.50, 0.49)	0.094		
Lens	−1.75 (−5.45, 1.94)	0.342			−0.50 (−2.42, 1.43)	0.604		
DR severity	7.14 (4.32, 9.97)	**< 0.001**	5.07 (1.02, 9.12)	**0.016**	2.01 (0.19, 3.83)	**0.031**	0.69 (−1.58, 2.97)	0.539
DR duration	−0.01 (−0.23, 0.21)	0.941			−0.01 (−0.11, 0.11)	0.965		
HbA1c	−1.09 (−2.48, 0.31)	0.121			−0.52 (−1.16, 0.11)	0.099		
BCVA	−6.77 (−17.81, 4.27)	0.227			−9.22 (−16.84, −1.60)	**0.018**	−4.62 (−11.05, 1.82)	0.157
SE	0.43 (−1.04, 1.89)	0.559			0.66 (−0.07, 1.39)	0.076		
IOP	0.02 (−0.57, 0.60)	0.952			−0.11 (−0.41, 0.19)	0.448		
Time	0.76 (0.06, 1.47)	**0.034**	0.73 (0.19, 1.27)	**0.009**	0.90 (0.25, 1.54)	**0.008**	0.87 (0.35, 1.39)	**0.002**

SVD, superficial vascular complex vessel density; DVD, deep vascular complex vessel density; DR, diabetic retinopathy; BCVA, best-corrected visual acuity; SE, spherical equivalent; IOP, intraocular pressure.

For DVD changes, univariate analyses identified age (estimate = −0.14, P = 0.008), DR severity (estimate = 2.01, P = 0.031), and time (estimate = 0.90, P = 0.008) were significant factors. In multivariate analyses, time (estimate = 0.88, P = 0.002) remained significant.

## Discussion

Various studies have explored on the effects of CaD on the retina of DR patients, but research specifically targeting its impact on the macular microvasculature has been absent until now. Our investigation using OCTA revealed an increase in macular SVD and DVD in DR patients following the initiation of CaD supplementation. Notably, macular SVD and DVD experienced a significant rise from baseline at 3 months post-CaD initiation, maintaining similar levels at 6 months. Moreover, DR severity was notably linked with changes in SVD, suggesting more pronounced alterations in SVD with CaD supplementation in patients with advanced DR stages.

This study documented significant increases across OCTA parameters, including SVD, SKSVD, DVD, SKDVD, and CVD over 6 months in DR patients under CaD supplementation. Previous research has thoroughly documented retinal microvasculature impairments in DR patients using OCTA [9–11]. For example, Sung et al. [9] observed that macular VD and perfusion density were significantly lower in patients with type 2 diabetes or DR than in normal controls. Similarly, Nesper et al. [11] found that DR patients, particularly those at an advanced stage, exhibited an enlarged foveal avascular zone area, decreased VD, and an increased percentage area of nonperfusion compared to healthy individuals. A significant reduction in SKSVD was also observed in the control group of our study. The observed VD increase following CaD supplementation in DR patients with compromised VD suggests a restoration of the macular microvasculature, potentially attributable to the prevention of oxidative stress, protection of the BRB, and reduction in retinal VEGF levels by CaD, highlighting the benefit of CaD for macular microcirculation in DR patients [12–14].

The significant increase in SVD and DVD relative to baseline observed by the third month did not further change significantly by the sixth month in Group 1. It suggests that the improvement in macular microcirculation induced by CaD might reach a plateau after a certain period, sustaining the level of improvement. Further investigations would benefit from more frequent examinations to ascertain the precise timing of this plateau effect. Regarding the CC, although a significant change in CVD was recorded over 6 months, pairwise comparisons failed to show a significant difference. Ashraf et al. [15] found that subfoveal choroidal thickness significantly increased from 316.08 ± 61.69 to 327.81 ± 58.03 μm following CaD treatment in DR patients. While CaD may improve choroidal blood circulation, its impact appears to be more pronounced in enhancing retinal microcirculation. Ultimately, the enhancement in macular microcirculation was sustained 6 months after initiating CaD supplementation.

In multivariate analyses, the severity of DR was significantly associated with changes in SVD, illustrating that patients with proliferative DR showed a more pronounced association with SVD increase compared to those with nonproliferative DR. Although previous research has highlighted the effectiveness of CaD in early-stage DR patients, reports on its impact in advanced stages are limited [5–7]. Our findings suggest that CaD may improve macular microcirculation even in advanced DR, potentially to a greater extent than in early-stage DR. This could be due to the more compromised macular microvasculature in proliferative DR, which might highlight the improvement effects of CaD. It is important to note, however, that all patients with proliferative DR in this study had previously undergone PRP, which may have influenced the observed CaD effects on macular microcirculation. Further prospective studies with larger cohorts are necessary to establish more definitively these observations.

This study has several limitations. First, its retrospective design inherently introduces some degree of bias. Additionally, although there was no significant difference in HbA1c levels between the two groups and no notable changes in medication were observed in our institutional records during the study period, we were unable to control for these factors. Therefore, we cannot rule out the possibility that changes in microcirculation may have been influenced by medications prescribed at other hospitals. This represents another limitation inherent to the retrospective design of our study. Second, the absence of diverse visual function tests precluded the analysis of the relationship between increased VD and visual function. Future prospective studies incorporating visual field tests, color-vision tests, and contrast sensitivity tests are essential to investigate the correlation between enhanced macular microcirculation and various visual functions more thoroughly. Third, the study did not account for various systemic conditions, such as body mass index, cholesterol levels, or hematologic status, which could potentially influence the outcomes. The strength of this study lies in its focus on the effects of CaD on macular microvasculature in DR patients, an area not previously reported. Another significant aspect is the inclusion of late-stage DR patients, offering insight into the utility of CaD beyond early-stage DR, as predominantly explored in prior studies.

In conclusion, CaD supplementation in DR patients led to significant increases in macular SVD and DVD after 6 months, with notable improvements observed within the first 3 months and maintained up to the sixth month. There was a significant relationship between changes in SVD and DR severity, indicating that the increase in SVD following CaD supplementation might be more substantial in patients with late-stage DR. Consequently, CaD intake is advantageous for the improvement of macular microcirculation in DR patients, applicable even in advanced stages of the disease. Further research is needed to clarify the effects of CaD on various visual functions in DR patients.

## Supporting information

S1 Data This file contains the complete dataset analyzed in the current study, including all variables used for statistical modeling. (XLSX)
